# SMN Requirement for Synaptic Vesicle, Active Zone and Microtubule Postnatal Organization in Motor Nerve Terminals

**DOI:** 10.1371/journal.pone.0026164

**Published:** 2011-10-12

**Authors:** Laura Torres-Benito, Margret Feodora Neher, Raquel Cano, Rocio Ruiz, Lucia Tabares

**Affiliations:** Department of Medical Physiology and Biophysics, School of Medicine, University of Seville, Seville, Spain; Brigham and Women's Hospital, Harvard Medical School, United States of America

## Abstract

Low levels of the Survival Motor Neuron (SMN) protein produce Spinal Muscular Atrophy (SMA), a severe monogenetic disease in infants characterized by muscle weakness and impaired synaptic transmission. We report here severe structural and functional alterations in the organization of the organelles and the cytoskeleton of motor nerve terminals in a mouse model of SMA. The decrease in SMN levels resulted in the clustering of synaptic vesicles (SVs) and Active Zones (AZs), reduction in the size of the readily releasable pool (RRP), and the recycling pool (RP) of synaptic vesicles, a decrease in active mitochondria and limiting of neurofilament and microtubule maturation. We propose that SMN is essential for the normal postnatal maturation of motor nerve terminals and that SMN deficiency disrupts the presynaptic organization leading to neurodegeneration.

## Introduction

SMN (Survival Motor Neuron) is a protein that participates in the assembly of small nuclear ribonucleoproteins (snRNPs) [1], [2], [3], [4]. SMN is encoded by two genes, *SMN1* that produces Full-Length SMN (SMN-FL), and *SMN2* that produces a truncated form of SMN (SMNΔ7) and a small amount of SMN-FL [5], [6], [7]. Spinal Muscular Atrophy (SMA), a severe autosomal recessive genetic disease in infants characterized by motor impairment and premature lethality [8], caused by mutations or loss of *SMN1* and retention of *SMN2* [9]. The severity of the disease depends on the amount of SMN-FL produced by *SMN2*.

SMN is expressed ubiquitously, nevertheless, abnormally low levels of SMN mainly affect motor neurons innervating proximal muscles, however the specific role of SMN in motor neurons remains unknown. Two main hypothesis have been postulated, first SMN deficiency is proposed to disturb relevant RNA processing in motor neurons [4], [10], [11], [12], [13], [14], [15], [16]; second, SMN is proposed to have specific functions in axon guidance and synapse maturation which are independent of its role in snRNP biogenesis [12], [13], [14], [16], [17], [18], [19], [20]. Interestingly, SMN is found not only in soma but also in axons [21] as well as at growth cones in cultured motor neurons [22]. SMN localizes in granules that are bi-directionally and rapidly transported [23], [24]. In fact, SMN has been shown to transport β-actin mRNA along the axon in embryonic neurons in culture [19], [25], [26].

A role for SMN in axonal guidance seems to be relevant in embryonic mouse motor neurons in culture, and in lower vertebrates SMA models [18], [19], [20], [23], [27], [28], [29], [XPATH ERROR: unknown variable "next".]. However, in SMA mouse models most neuromuscular junctions (NMJs) are normally innervated at early postnatal ages [31], [32], [33], suggesting that in higher vertebrates *in vivo* neurite outgrowth and path finding occur appropriately. On the other hand, in mouse models of SMA a series of alterations at the level of the NMJ have been reported, ranging from neurofilaments accumulation [32], [34], [35], [36] and immaturity of the postsynaptic site [32], [33], [35], [36] to reduced neurotransmitter release [35], [36], [37]. Some of these alterations have been proposed to be due to a deficiency in axonal transport [38]. New findings supporting this hypothesis show that polymerized tubulin levels are decreased in sciatic nerves from SMA-like mice, while stathmin, a microtubule-destabilizing protein, is upregulated in this mouse model [39].

Here, to better understand the alterations of neurotransmission at the NMJ we undertook a comprehensive functional and morphological study in a SMA mouse model (SMNΔ7, [40]). We have explored the organization of synaptic vesicles (SVs), active zones (AZs) and mitochondria within the terminal at P7 and P14, the time points at which functional alterations have already been reported [36]. In addition, we investigated the relationship between SVs and the cytoskeleton (neurofilaments, actin and microtubules). These observations have been combined with electrophysiological estimates of the size and kinetics of the readily releasable pool (RRP) of SVs. We present evidence that SVs and AZs are clustered, the RRP size and the mitochondrial pool size are reduced, and microtubules are highly disorganized in SMN-deficient presynaptic terminals. Taken together these results suggest that SMN is essential for the normal postnatal organization and maintenance of the presynaptic motor terminal.

## Results

### Synaptic vesicles are anomalously clustered and less abundant in SMN*-*deficient motor terminals

To get insight into the postnatal presynaptic maturation process in SMN-deficient terminals, we first examined SVs organization in young mice (P7 and P14) in the TVA muscle, one of the most affected in this disease [33], 36. We explored both the total area of the terminal covered by SVs and the SVs spatial organization using antibodies against the vesicular ACh transporter (VAChT), conjugated with fluorescent secondary antibodies.

**Figure 1:**
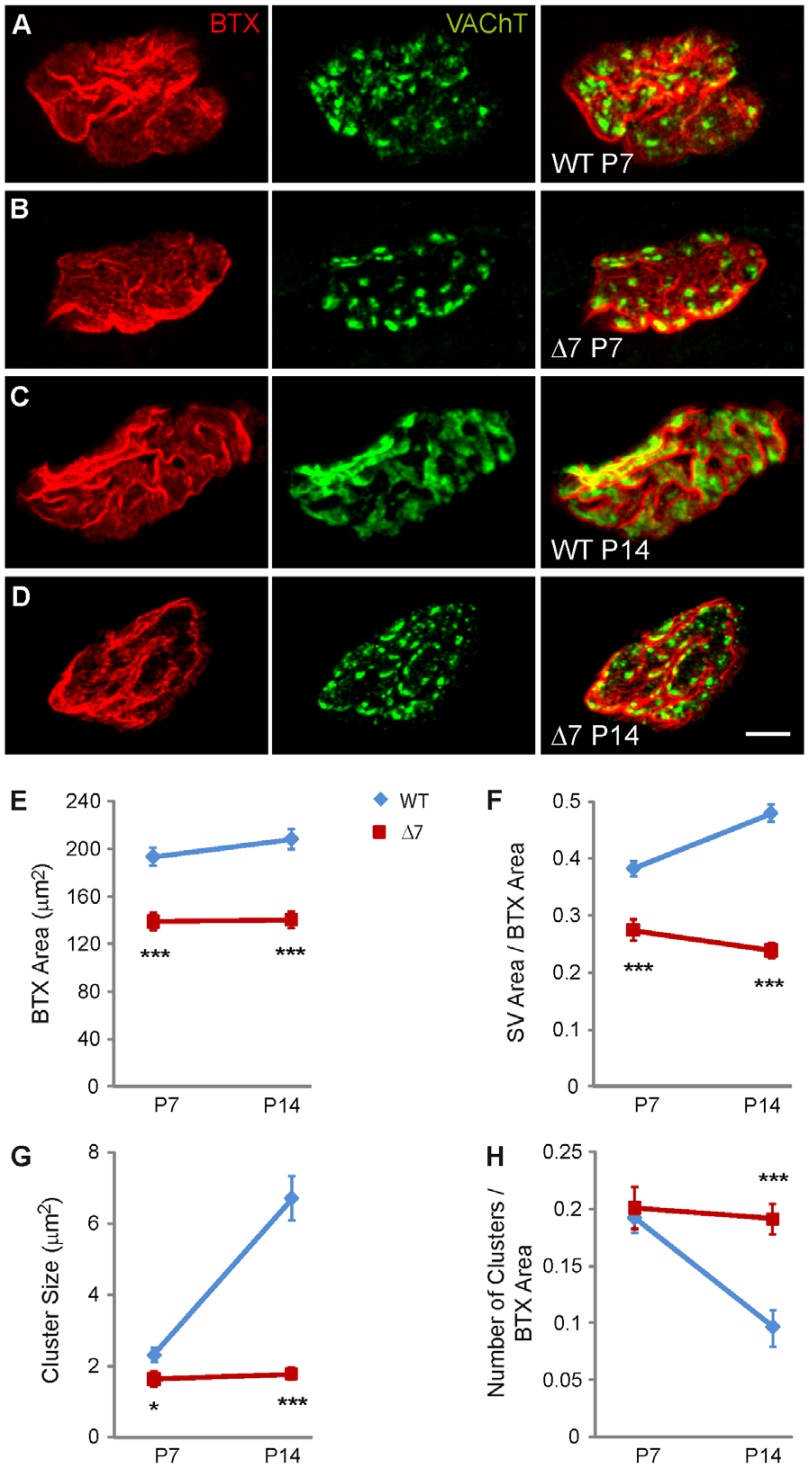
Synaptic vesicles in motor terminals of SMNΔ7 mice are reduced and anomalously distributed. Representative *en face* views of NMJs from the TVA muscle stained with BTX-Rho (red), which binds specifically to postsynaptic AChRs, and anti-VAChT (green), which labels synaptic vesicles. Images are Z-stack projections. **A & B.** Terminals of WT and SMNΔ7 mice, respectively, at P7. **C & D.** At P14, the postsynaptic apparatus and SVs appeared less mature in mutants (D) than in WTs (C), and SVs in mutants appeared in compact clusters while they distribution in WT terminals were predominantly diffuse. **E.** Mean postsynaptic areas were significantly smaller in mutants than in WT terminals, both at P7 and P14. **F.** The area of VAChT stained vesicles (normalized to the postsynaptic size) was smaller in SMNΔ7 than in WT motor terminals. **G**. The mean size of vesicle clusters in SMNΔ7 was smaller than in WT at P7 and P14. **H**. The mean number of SV clusters was larger in SMNΔ7 terminals at P14. Scale bar: 5 µm; *: P<0.05; **: P<0.005; ***: P<0.0005.

In wild-type (WT) mice, at the beginning of the NMJ postnatal maturation period (first week of life), SVs were observed to be grouped in small clusters (Fig. 1A), but by the second week, as WT NMJs matured, SVs appeared to spread out and covered larger areas of the terminal (Fig. 1C). In SMN-deficient terminals, however, SVs remained clustered during this period (Fig. 1 B & D).

As the size of the NMJ was smaller in mutants (Fig. 1E), the areas covered by SVs were normalized to the area of the postsynaptic terminal. The total surface area of the terminal covered by SVs was ∼30% smaller in SMN-deficient terminals (*n* = 37 NMJs from 3 mice) than in WTs (*n* = 38 NMJs from 3 mice) at P7 (Fig. 1F; P<0.0001). At P14, this difference increased to almost 50% (Fig. 1F; P<0.0001; WT: *n* = 52; SMNΔ7: *n* = 58 terminals).

In addition, the size of SV clusters was smaller in mutants than in WTs, both at P7 (∼30%; P = 0.02) and P14 (75%; P<0.0001) (Fig. 1G). Remarkably, in mutants no change in cluster size, or in the number of cluster per terminal, took place during the second week of life, while in littermate controls the clusters size increased, and the number of clusters diminished (Fig. 1G & H).

To test whether the clustering of SVs observed in the TVA muscle of SMA mutants with anti-VAChT antibodies were also seen with another vesicle marker, a double staining of SVs using antibodies against VAChT, and the synaptic vesicle protein 2 (SV2) was performed. The vesicular fluorescence patterns observed with anti-VAChT (green) were also seen with anti-SV2 (blue), both in WT, and in mutant terminals (Fig. S1). Quantitative analysis of the immunofluorescence signals indicated that the colocalization indexes of these two markers were ∼0.9 in both types of mice (Fig. S1). These data corroborate the smaller size of the clusters in SMA mutant terminals, and rule out the existence of an atypical vesicle population devoid of the vesicular transporter.

### The synaptic vesicle pools and the vesicle release probability are decreased in SMA

Electrophysiological recordings have shown that quantum content of evoked End Plate Potentials (EPPs) is reduced in SMA terminals [35], [36], [37]. This, combined with the reduced area of immunolabeled SVs (Fig. 1), led us to examine the size of the RRP of SVs (docked vesicles) by functional analysis.

**Figure 2:**
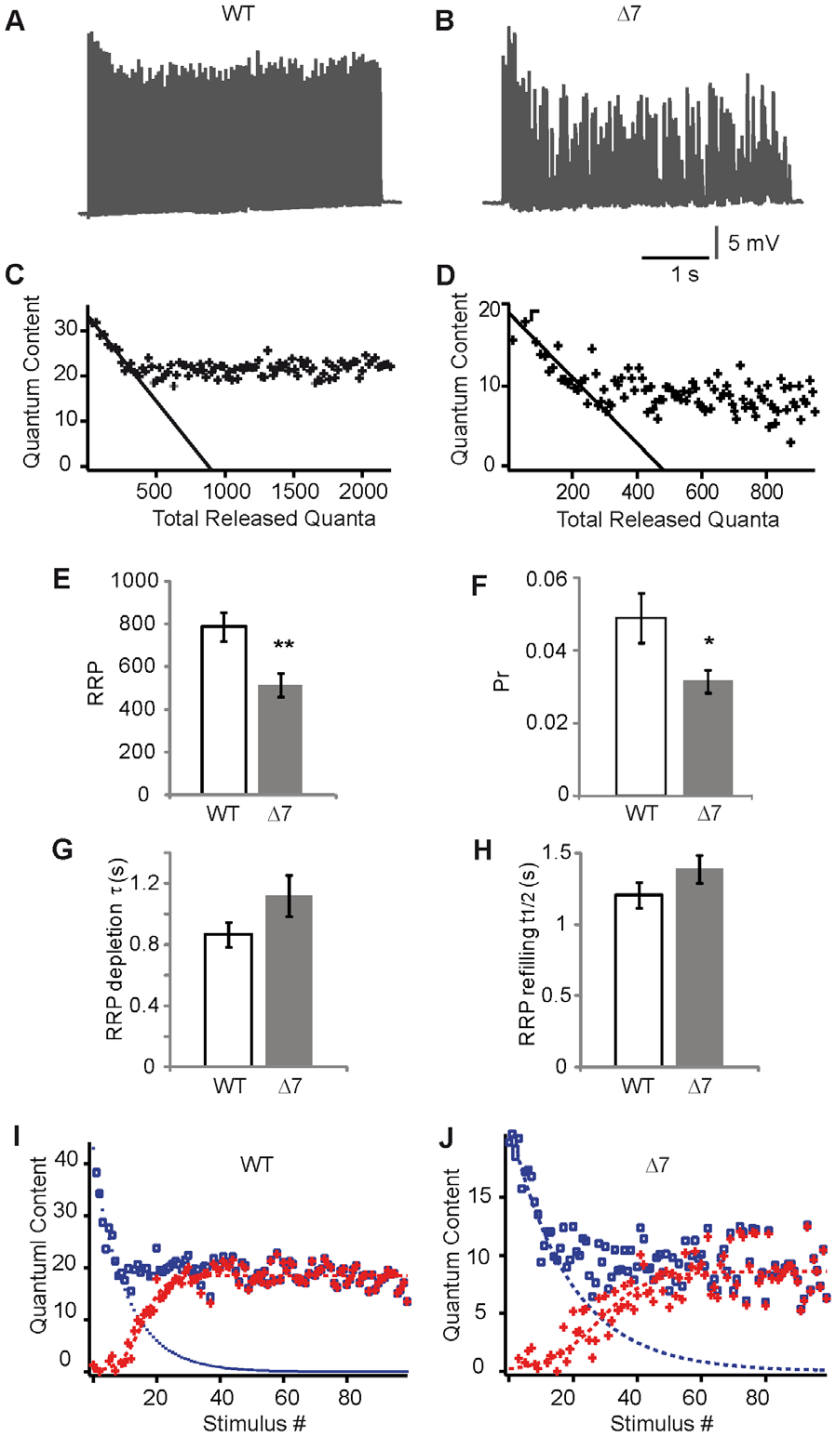
RRP and Pr are reduced in motor terminals from the SMNΔ7 TVA muscle. **A & B**. Representative traces of EPPs during 5 s trains of stimuli at 20 Hz in WT and SMA mice (P14), res pectively. **C & D**. Examples showing the technique for the estimation of RRP size by the x-intercept of the fitted points. **E–H.** Mean RRP size, vesicle release probability, depletion time constant, and refilling half time, in WT and SMNΔ7 terminals. **I & J**. Representative examples for the estimation of the kinetic properties of the recruitment process in WT and SMNΔ7. * P<0.05; **: P<0.005.

Figure 2 shows representative WT (A) and SMNΔ7 (B) EPPs evoked by a stimulus train (20 Hz, 5 s) recorded in the TVA muscle. During the initial part of the train, peak EPP amplitudes follow a characteristic pattern consisting of a small amount of facilitation followed by an exponential decline, corresponding to the depletion of vesicles in the RRP 41,42. Normally, these phases are followed by a steady-state period (plateau) of matched balance between vesicle consumption and refilling of release sites (Fig. 2A). In SMA mutant terminals, however, rarely was there a plateau and, instead, EPP amplitudes were highly variable (Fig. 2B), suggesting the inability of mutant synapses to adjust their neurotransmitter release to a constant rate or their low release probability.

To examine the size of the RRP, the quantum content for each stimulus was calculated by dividing the corrected mean EPP value [43] by the mean miniature (mEPP) size. Then, the RRP was estimated, by plotting quantum content against accumulated quantum content (Fig. 2C & D), and drawing a straight line through the declining phase [41]; the x-axis intercept of the fitted points gives an estimate of the RRP size, with the assumption that during the first 10–15 shocks most of the release comes from the RRP. The mean RRP size was significantly reduced (by 35%) in SMA mice (SMA: 515±54; *n* = 22 terminals, 6 mice; WT: 787±67; *n* = 23 terminals, 5 mice; P = 0.003) (Fig. 2E). The vesicle release probability (Pr) was also decreased in mutants (0.049±0.007, *n* = 23 in 5 WT mice, and 0.032±0.003, *n* = 22 in 6 mutant mice; P = 0.026) (Fig. 2F), suggesting that the reduced quantum content [36] was due to a reduction in both the RRP size and the Pr.

In addition, the total number of quanta released during the 5 second train was about 58% less in mutants (955±88 quanta, *n* = 16) than in WT littermates (2258±355 quanta, *n* = 16) (P = 0.002), due to the lower quantum content and to a higher level of synaptic depression in mutant terminals, indicating that it also existed a reduction in the recycling pool (RP) of SVs.

### Mobilization of vesicles to the readily releasable pool is not altered in SMA

The refilling of the RRP takes place by mobilization of new vesicles from the recycling pool (RP) and/or by the recycling of vesicles from nearby regions after undergoing a previous round of exocytosis. A variation of the Elmqvist & Quastel method [41] was used to estimate the kinetics of the RRP depletion and refilling (see methods and [42]). When we compared the time constant of the RRP exhaustion in WT and mutants (discontinuous blue line in Fig. 2I & J), no significant difference was found (WT: 0.9±0.1 s; *n* = 16 terminals, 3 mice ; SMNΔ7: 1.1±0.1 s; *n* = 16 terminals, 4 mice; P = 0.12) (Fig. 2G); similarly, the mean half-time taken for vesicles to refill the RRP (discontinuous red line in Fig. 2I & J), was not significantly different in mutants (1.4±0.1 s, *n* = 16) than in WTs (1.2±0.1, *n* = 16; SMNΔ7; P = 0.17), (Fig. 2H). These data suggest that the depletion kinetics of vesicles at AZs, and the availability of new vesicles for successive rounds of exocytosis were not slowed in mutants.

### Mitochondria are diminished but well co-localized with synaptic vesicles in SMA

Some of the roles of presynaptic mitochondria are to provide ATP for synaptic transmission, ion pumps, and assembly of actin cytoskeleton involved in the clustering of SVs and mitochondria themselves. Another role of mitochondria is regulation of intraterminal Ca^2+^ levels, providing an especially important buffering action during trains of action potentials. Therefore, a reduction in the number of mitochondria, alteration in their spatial distribution, or impairment of their functional capacities, may decrease their Ca^2+^ buffering capability and produce toxic Ca^2+^ overloads. To assess whether an alteration in mitochondria spatial distribution, or density was present in SMN deficient terminals, the TVA muscle was incubated with mitotracker (400 nM), a cell-permeant probe that is sequestered by functioning mitochondria and retained during cell fixation [44].

**Figure 3:**
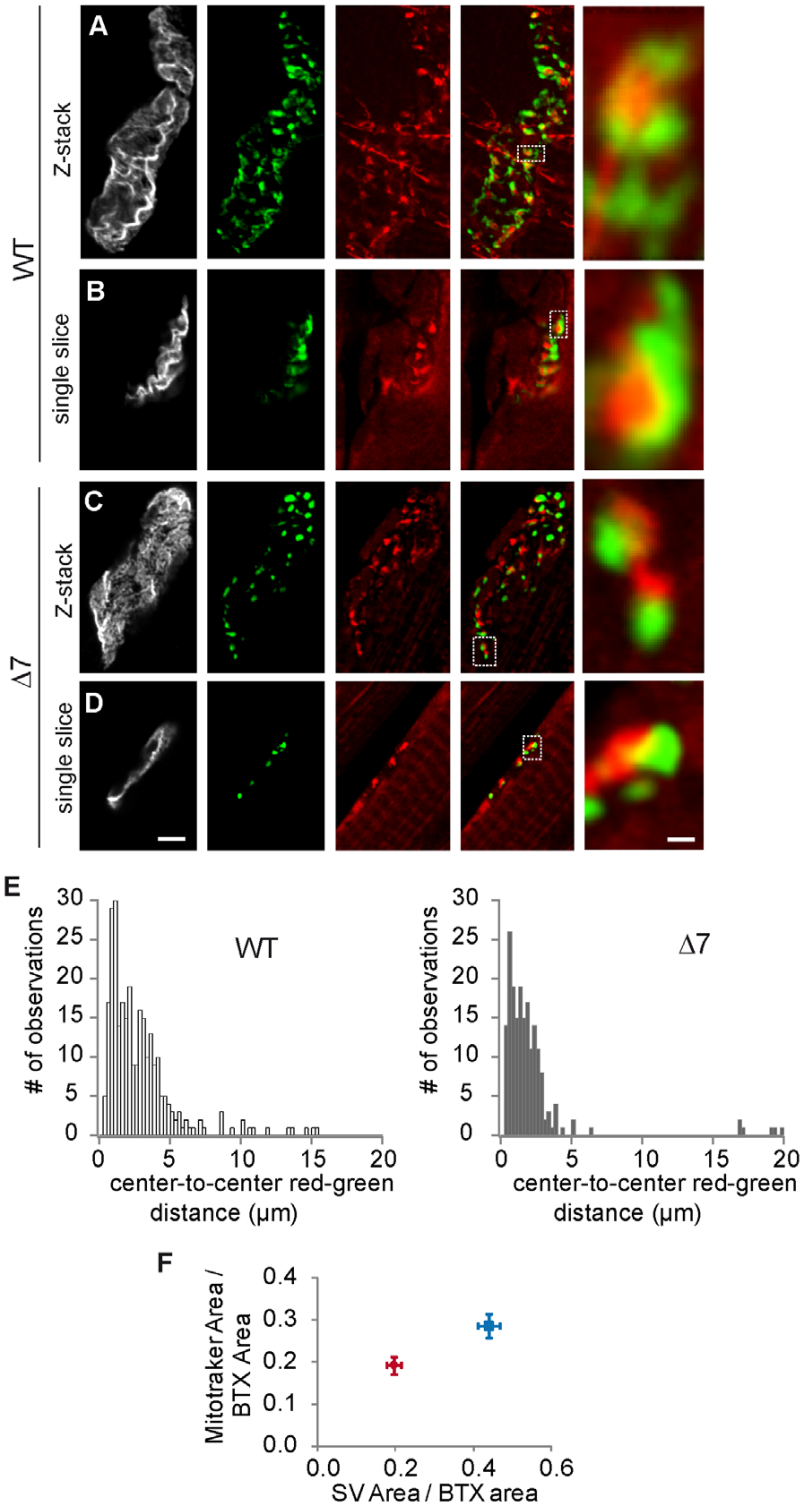
Mitochondrial density in SMA presynaptic terminals is reduced. **A–D**. The upper panels (A & C) show Z-stack projections while the lower panels (B & D) show single confocal sections. The staining from left to right are: BTX-A647 (grey), SVs (green), mitotracker (red), merge of SV and mitochondria. Data are from the TVA muscle (P14). **E.** Nearest neighbor distributions of SV clusters and mitochondrial regions (calculated from their respective center of mass), in WT and mutants terminals. **F.** Relationship between mean areas covered by SV and mitochondria in WT (blue symbol) and SMNΔ7 (red symbol) terminals. Scale bars A-D: 5 µm; insets: 600 nm.

Figure 3A-D (central panels) shows representative examples of the distribution of mitotracker labeled mitochondria in motor terminals. Quantification of the number of mitotracker spots per terminal showed no significant differences between WT and mutants (WT: 22.9±2.1 spots/terminal, *n* =  18 terminals; mutant 23.5±3.1, *n* = 19 terminals) (P = 0.9). The total area stained by mitotracker per terminal, however, was about half in mutants (19.4±2.7 µm^2^; *n* = 20) as compared to WT terminals (38.8±4.9 µm^2^; *n* = 22; P = 0.002). Normalization of these areas to postsynaptic surface area also gave significantly smaller values in mutants (WT: 0.29±0.03; mutant: 0.19±0.02; P = 0.01). This difference was mainly due to the smaller size of the mitotracker spots (WT: 1.72±0.36 µm^2^, *n* = 360 spots; mutant: 0.89±0.1 µm^2^, *n* = 214 spots. P = 0.03) in mutants.

Next, we explored the spatial relationship between mitotracker spots and SV clusters. In most cases, as in the illustrated examples, mitochondria (red) appeared near SV clusters (green) in both WT (Fig. 3A & B) and mutant terminals (Fig. 3C & D). Moreover, in many cases a core of mitochondria was clearly surrounded by a SV rim (Fig. 3A–D, right panels), reminiscent of the donut-like structures described in the calyx of Held [45], [46]. Quantification of this co-clustering was done by measuring the nearest-neighbor distances between SV clusters and mitochondria spots. Nearest-neighbor distances were determined by fitting each of the red and green spots to a 2D Gaussian distribution, and using the peak (x,y) of each distribution. The results in nine mutant terminals (202 measurements) and 13 WT terminals (282 measurements), showed that in mutants SVs and mitochondria were, as in controls, closely associated (Fig. 3E). Even more, the percent of red-green spots nearest distances within 1 µm were larger in mutants (41%) than in WT (31%) (P = 0.0006), as expected for the smaller sizes of SV and mitochondria cluster in mutants (Fig. 3F).

Taken together, these results show that in mutant terminals the close spatial relationship between mitochondria and SV clusters was not lost, but revealed a reduction in the amount of functional mitochondria.

### Active zones are reduced and clustered in SMA

Given the reduction on the RRP size found in mutants (Fig. 2), we wondered whether the number of AZs per terminal was reduced in comparison with littermate controls. To identify AZs, an antibody against bassoon, a scaffolding protein of AZs [47], was used in the TVA muscle.

**Figure 4:**
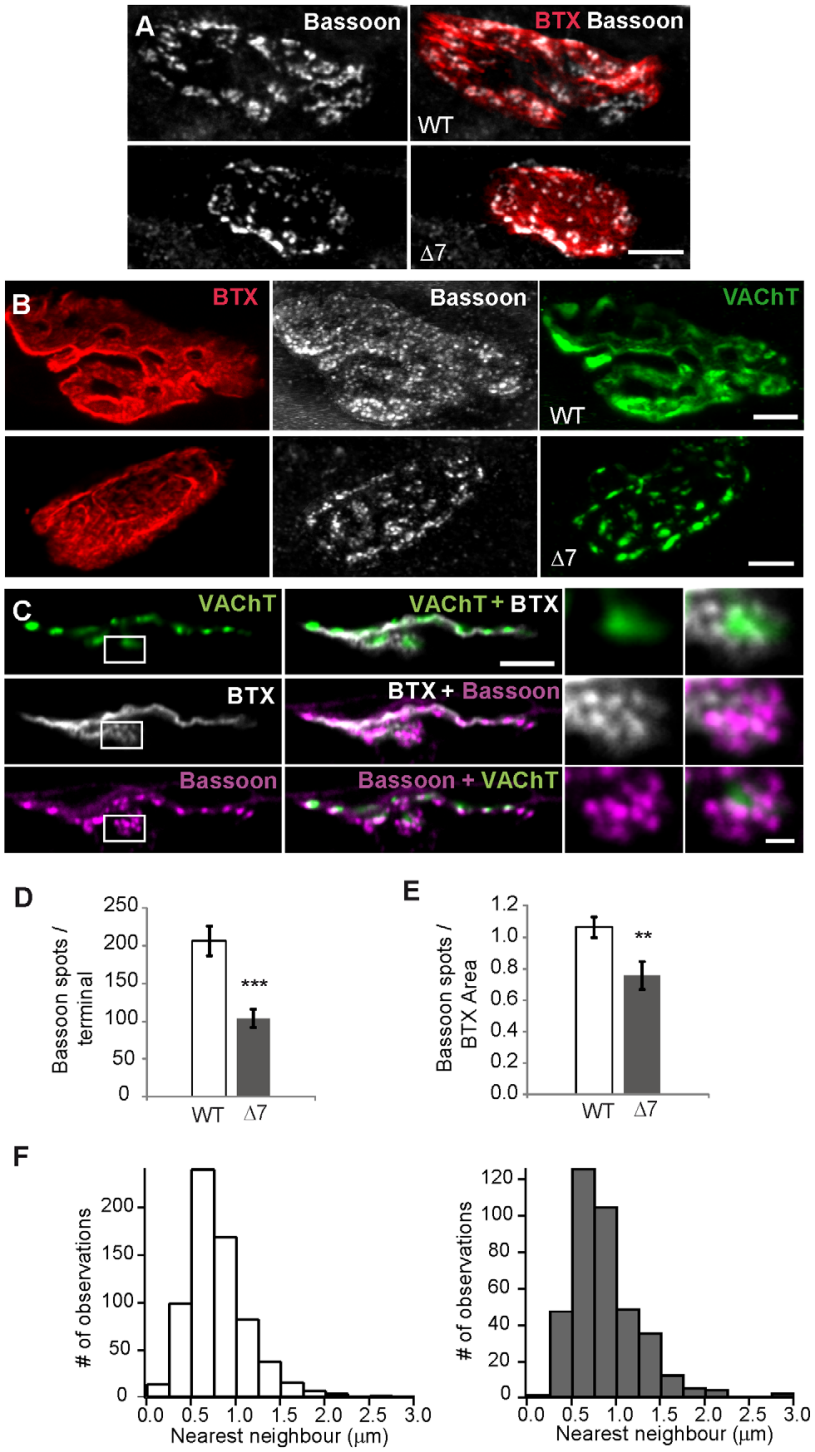
AZs in SMA motor terminals are reduced but well associated to SV clusters. **A.** NMJs at P7 labeled with anti-bassoon (left), and BTX-Rho (right), in a WT (upper panels) and mutant (lower panels) muscle. In WT bassoon spots are abundant throughout the terminal, while in the mutant spots are scarce. **B.** NMJs at P14, bassoon and postsynaptic receptors labeled as in A, SVs labeled with anti-VAChT (right), in a WT (upper panels) and mutant (lower panels) TVA muscle. In WT bassoon spots appear distributed evenly throughout the terminal, while in the mutant spots are clumped in groups that co-localize with SVs. **C.** Planar projection of a mutant NMJ stained with anti-bassoon (magenta), BTX (grey) and anti-VAChT (green) showing clustering of SVs and bassoon spots. **D–E**. Mean number of bassoon spots per terminal (left graph), and per µm^2^ (right graph) in WT (*n* = 15) and SMNΔ7 (*n* = 20) fibers. **F**. Distribution of bassoon spots interdistance (nearest neighbors) in three WT (*n* = 694) and three SMNΔ7 (*n* = 399) fibers. Scale bars: 5 & 1 (insets) µm. ** (P = 0.009); *** (P = 0.0002).

Typically, AZ number and distribution change during the postnatal maturation period. Fig. 4A shows, at P7, two typical examples of the lower abundance and differential distribution of bassoon spots in mutant (lower panel) compared with WT (upper panel) terminals. At P14, these apparent differences were even clearer. In the examples shown in Figure 4B the total number of bassoon spots counted in each nerve terminal were 273 in the WT (central upper panel) and 139 in the mutant (central lower panel), which resulted in 1.29 and 0.69 bassoon spots/µm^2^, respectively. On average, at this age, the number of bassoon spots per terminal was 208±20 (*n* = 20), in WT and 102±12 (*n* = 15), in mutants (Fig. 4D) (P = 0.0002), and their density 1.1±0.1 bassoon spots/µm^2^ in WT, and of 0.7±0.1 in mutants (Fig. 4E) (P = 0.009). In addition, while in WTs bassoon spots appeared regularly distributed within the terminal (cf., Fig. 4B, central panel), in the mutants spots were apparently grouped and co-clustered with SVs (cf., Fig. 4B, lower central and right panels). Figure 4C illustrates bassoon spots in another mutant terminal, now in a planar projection; bassoon spots were clearly associated with SV clusters and, in some cases, a SV cluster was surrounded by a group of bassoon spots (Fig. 4C, inset).

The spatial distribution of the bassoon spots was quantified by nearest neighbor analysis performed on whole terminals. Fig. 4F shows the distance between bassoon spots (centers of 2D gaussian fits) from three WT (left) and three mutant (right) terminals. In both, the shape of the distribution were quasi normal-shaped. The average nearest neighbor separation in WTs (876±27 nm; *n* = 694 particles) was not significantly different (P = 0.27) to that in mutants (921±29 nm; *n* = 399 particles). These results indicate that in mutants AZs were regularly spaced but they were missing in some areas, following the anomalous clustering of SVs within the terminal.

### Neurofilament accumulation follows synaptic vesicle reduction

NF accumulation is a hallmark of many neurodegenerative diseases [48], including SMA mouse models [32], [33], [34], [35], [36]. Therefore, to better understand the pathophysiology of this process the spatial distribution of the NFs, and the relationship between NFs and the clusters of SVs were studied in the TVA muscle during the first and second week of postnatal life.

**Figure 5:**
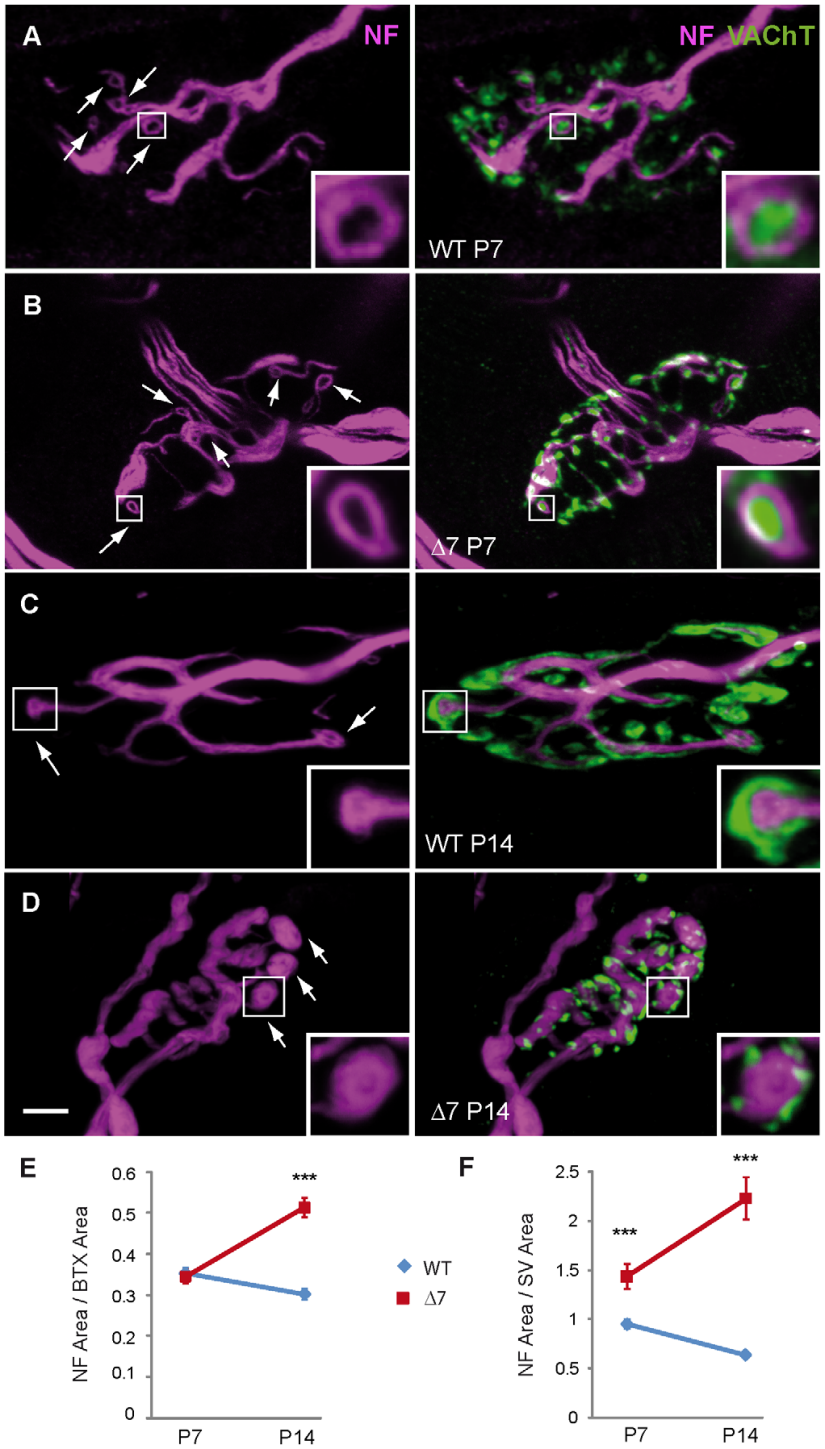
NF accumulation in SMA terminals occurs at a late stage of the disease. **A–D**. Left panels: NF staining (magenta) showing ring-like structures. Discontinuous lines (Panel B) mark dim NF labeling. Right panels: Merged images showing the spatial relationship between SVs and NFs. Note that at P7 SV clusters were near NFs, either inside the loops (arrows) or along their paths (A & B), while SVs were around the compact ring-like structures, at P14. Data are from TVA muscles of WT and SMNΔ7 mice. Scale bar: 5 µm. Images are Z-stack projections. **E.** NF area, normalized to postsynaptic area, in P7 and P14 WT and mutant terminals. **F**. Surface ratio between NFs and SVs in WT and mutants at both ages. *: P<0.05; **: P<0.005; ***: P<0.0005.

Interestingly, at P7, SV aggregates localized along the NF trajectory, both in WT and mutant terminals (Fig. 5A & B). In many cases, NFs ended in loop-like structures about 1.35 µm in diameter (arrows), which hosted clusters of SVs (Fig. 5A & B, insets). These structures, which are characteristic of cytoskeletal immaturity [49], [50], were equally frequent in mutant and WT terminals at this age (mean number of loops/terminal: 4.4±0.6, *n* = 38 versus 4±0.4, *n* = 37; P = 0.45).

At P14, the spatial localization of SVs along the NF trajectory persisted in mutants (Fig. 5D) while it was less obvious in WT terminals (Fig. 5C). Also, at this age the loops were rare (0.9±0.2 loops/terminal, *n* = 52) and small (1.2±0.1 µm in diameter, *n* = 20) in WT terminals (Fig. 5C, inset), while they were significantly more frequent (2.9±0.3 loops/terminal, *n* = 48) (P<0.0001), and enlarged (1.7±0.1 µm diameter, *n* = 20) (P = 0.0014) in mutants (Fig. 5D, inset).

The mean area of the terminal occupied by NFs was 1.7-fold higher in mutant than in WT terminals at P14 (P<0.0001; *n* = 38) but was not significantly different at P7 (Fig. 5E). Given the apparent reduction in SVs at this age, the surface ratio between NFs and vesicles was ∼3.5-fold larger in mutants than in controls, while it was only 1.5-fold larger at P7 (Fig. 5F).

These results indicate that NF postnatal maturation was interrupted in mutants and, in addition, suggest that the amount of NF protein was not down regulated as in controls, resulting in their accumulation during the second postnatal week. The data also show that NF accumulation followed the decrease in SVs.

### F-actin and synaptic vesicles are closely associated in SMA

Filamentous actin (F-actin) is a prominent cytoskeletal element in nerve terminals. The F-actin-based network [51] may participate in creating a scaffold for SV clustering, and/or in supporting ordered vesicle mobility. In addition, it has been suggested that F-actin anchors synaptic vesicles to AZs by a labile link formed with synapsin, a vesicle protein [52], [53].

SMN deficiency produces defects in beta-actin mRNA axonal transport, and a decrease in actin protein content in growth cones of motoneurons in culture [19]. We explored F-actin content and distribution in presynaptic TVA motor terminals of SMA mutant mice. F-actin was revealed by binding to fluorescent Phalloidin-Alexa 647, which binds to all isoforms of F-actin but not to monomeric actin [54]. In addition, to gain insight into the role of F-actin in the organization of SVs, the distributions of F-actin relative to SVs were also examined.

**Figure 6:**
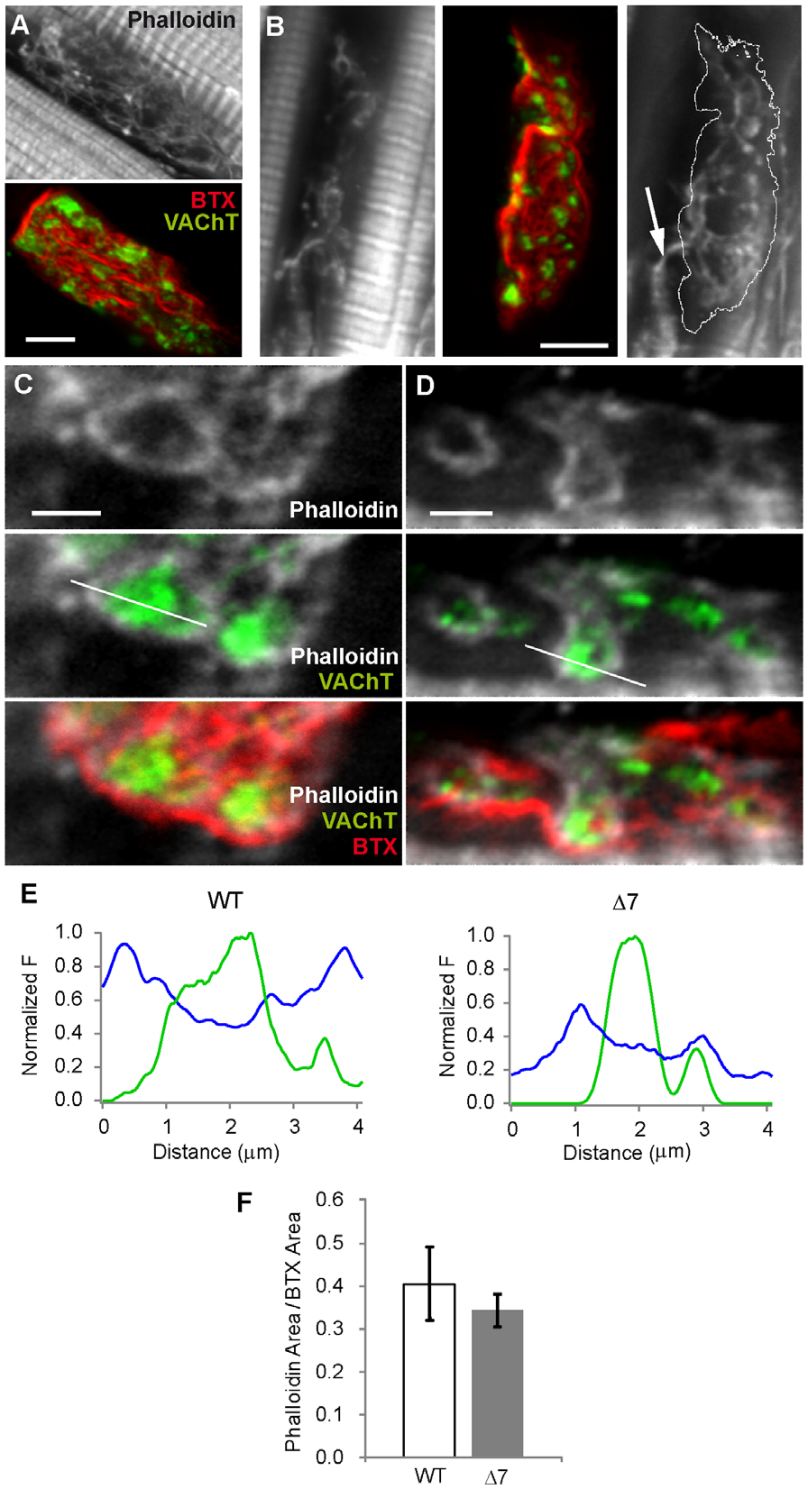
F-actin and SVs are closely associated in SMA motor terminals. Data are from the TVA muscle (P14) **A–B.** Representative examples of the distribution of F-actin (grey) in a WT (A) and a mutant (B) terminal. The same terminals are also shown stained for SVs (green) and postsynaptic receptors (red). Arrow in the right panel of B points to actin in the axon. **C–D.** Phalloidin (grey), SVs (green), and AChRs stained with BTX-Rho (red), showing the organization of actin around SV clusters. Image in C is from a SMNΔ7 terminal and in D from a WT terminal. **E.** Intensity profiles across actin loops and SV clusters showing the reduced diameter and thickness of the loop in mutants. **F.** Phalloidin areas in WT and mutant terminals were not significantly different (P>0.05). Scale bars: A & B: 5 µm; C & D: 2 µm.

F-actin was localized in the sub-plasmalemmal region and deep into the cytoplasm forming a thin network. Figure 6A shows a WT terminal with F-actin labeled with phalloidin (upper panel), SVs labeled with VAChT antibodies (green), and postsynaptic receptors labeled with BTX-Rho (red, lower panel). In Figure 6B a typical terminal from a mutant mouse is shown (left panel, phalloidin image; central panel, anti-VAChT and BTX-Rho merged). In some cases, F-actin was also clearly visualized in the axon (Fig. 6B, arrow in right panel). In WT and mutant mice, F-actin filaments run parallel to the major axis of the terminal and SV clusters are positioned along them.

The association between SV clusters and actin could be better visualized in transverse single confocal slices. Figure 6C (WT) and 6D (mutant) show two representative examples showing F-actin surrounded SV clusters forming ring-like structures, similar to actin rings described in the lamprey reticulospinal giant synapse [55]. Interestingly, the diameter of F-actin rings surrounding SVs were apparently smaller in mutant than in WT terminals, as could be appreciated by their respective line intensity profiles across SVs and phalloidin loops (Fig. 6E). However, these structures were not visualized frequently enough for statistical analysis. Thus, the percentage of the terminal area occupied by phalloidin, normalized to the postsynaptic area, was quantified (Fig. 6F). In WT mice, although the area of the terminal covered by phalloidin was apparently larger than in mutants (WT: 41±9%, *n* = 6 terminals; mutants: 34±4%, *n* = 6 terminals), this difference did not reached statistical significance (P = 0.5).

### Severe alteration of microtubules organization in SMA

Microtubules provide rails for the transport of organelles by means of microtubule-associated motor proteins. To examine the microtubule organization in the nerve terminal of controls and SMNΔ7 mutants an acetylated tubulin antibody, which marks polymerized tubulin, was used.

**Figure 7:**
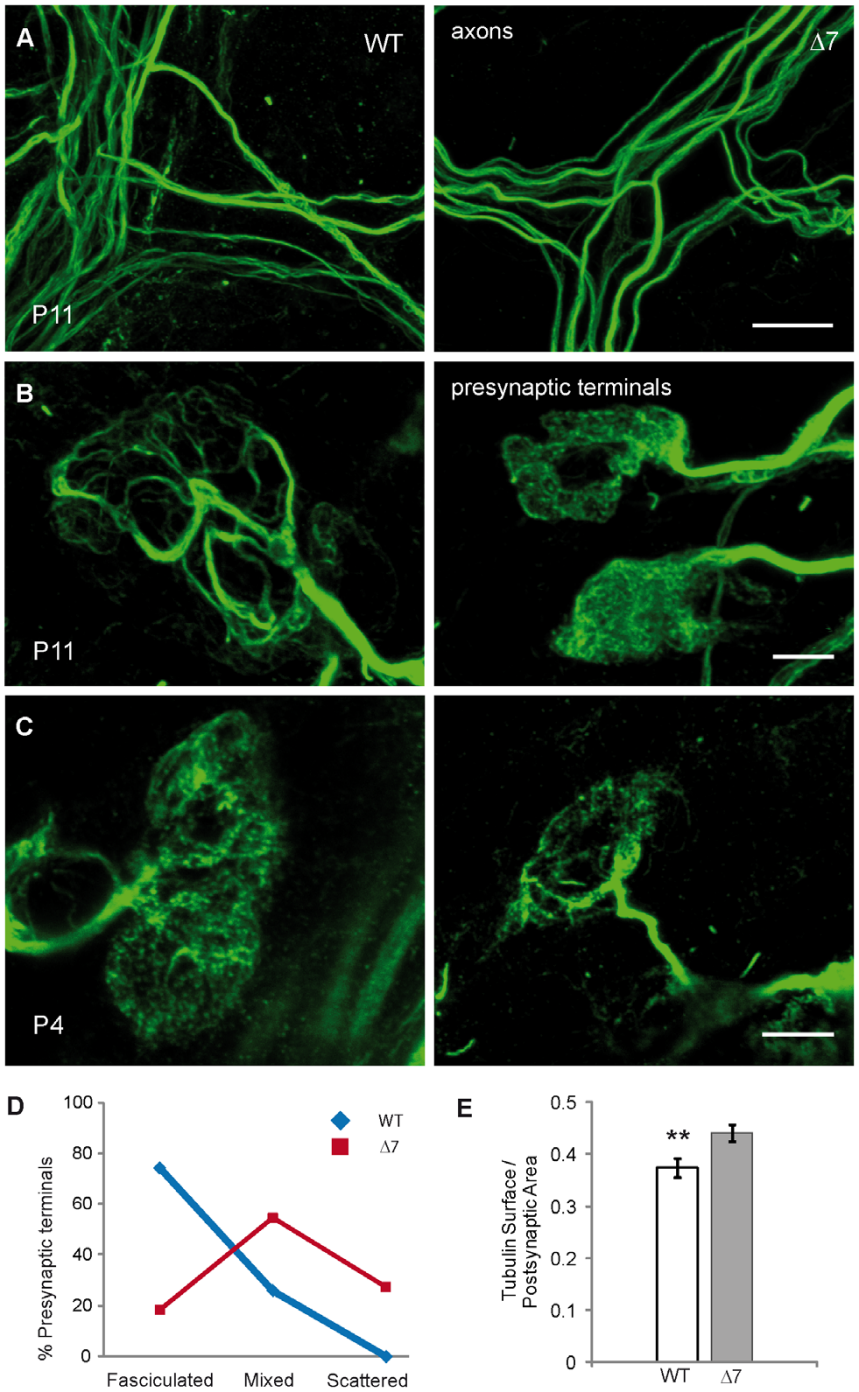
Microtubules are immature and more abundant in SMA presynaptic terminals. **A.** Representative images from WT (left) and SMNΔ7 (right) axon microtubules in TVA muscles at P11 apparently showing no alterations. Scale bar: 10 µm. **B–C.** Typical distribution of microtubules in WT and mutant nerve preterminals, at P11 (B) and P4 (C). Scale bars: 5 µm. **D.** Percentage of presynaptic terminals showing microtubules in a fasciculated-like, scattered-like, or mixed pattern, at P11. **E.** Microtubule area in the presynaptic terminal, normalized to the postsynaptic surface, was significantly larger in SMNΔ7 than in WT mice. **: P<0.005.

In axons from the TVA muscle, no difference in tubulin distribution between WT and mutant mice was observed at P9-P11, suggesting that axon guidance and fasciculation apparently were not affected in SMNΔ7 mutant mice (Fig. 7A). In the presynaptic terminals, however, the organization of polymerized tubulin differed in WT and mutant mice (Fig. 7B). In most WT terminals, microtubules appeared grouped in thick and thin bundles (left panel). Nevertheless, in most mutants microtubules appeared scattered all over the terminal, instead of fasciculated, and with a punctuate appearance (right panel). We quantify the percent of terminals containing branched-like, scattered-like, and mixed patterns (Fig. 7D). In WT mice, the branched-like pattern was the most frequent (74%), and no scattered-like ones were observed. In mutants, on the other hand, the majority of terminals showed a mixed pattern (∼54%), and only 18% showed a normal branched pattern.

Quantification of the tubulin stained area per terminal gave no significant differences between controls and mutants (81.09±3.81 µm^2^, *n* = 32 terminals, vs. 76.49±3.72 µm^2^, *n* = 33; P = 0.39). However, when these values were normalized to the postsynaptic areas, mutants showed to have a relative greater amount of microtubules than controls (0.44±0.02 vs. 0.37±0.02), (P = 0.0083) (Fig. 7E).

To explore whether the anomalous organization of microtubules in SMNΔ7 terminals was due to the immaturity of the presynaptic terminal, we looked at the distribution of microtubules at an early age (P4). Interestingly, microtubules appeared scattered and punctuated in WT terminal at this age (Fig. 7C, left panel), similar to the pattern in mutants from P4 to P11 (Fig. 7 B & C, right panels), suggesting a defect in the rearrangement of microtubule architecture during the postnatal period in mutants.

**Figure 8:**
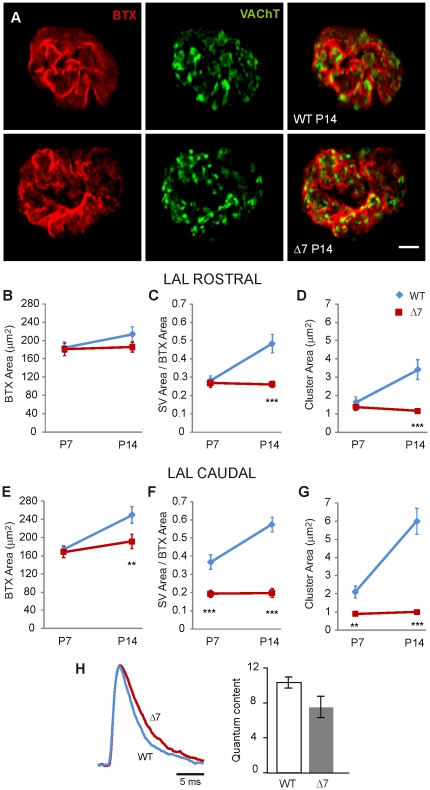
Selective alterations of SVs in motor terminals from the LAL muscle. **A.** Representative images of pre- and postsynaptic characteristics of NMJs in the LAL muscles of WT (upper panels) and SMNΔ7 (lower panels) mice at P14. Postsynaptic terminals were labeled with BTX-Rho (red) and presynaptic vesicles with antibodies against VAChT (green). Note the clustering of SVs despite the mature appearance of the postsynaptic side in the SMNΔ7 terminal. Scale bar: 5 µm. **B–G.** Mean postsynaptic area (B & E), SV area (C, F), and cluster area (D, G) in SMNΔ7 and WT terminals, from rostral (B–D) and caudal (E–G) divisions of the muscle. Note that the area of vesicles and the cluster sizes were not significantly different in the rostral part of SMNΔ7 terminals at P7, while these parameters were significantly reduced at P14. However, in the caudal part SVs deficiencies in SMNΔ7 terminals were evident since P7 (*n* = 16 WT terminals and *n* = 15 SMNΔ7 terminals). *: P<0.05; **: P<0.005; ***: P<0.0005. **H.** Representative EPP traces of WT and mutant terminals (left panel). Quantum content (right panel) was not significantly different in WT and mutants terminals (P = 0.07).

The above results, together with the other presynaptic defects in the TVA muscle, raised the question of how specific this phenotype is. Therefore, we explored the NMJs in the LAL, a muscle in which SMNΔ7 postsynaptic sites have been described to mature almost at the same pace as in control mice [33], with the exception of NMJs in the caudal band which are more vulnerable to low SMN levels [33], [36]. In the rostral band, at P7, postsynaptic area (Fig. 8 B), SV content (Fig. 8C), and SV cluster size (Fig. 8D) were not different in WT and mutant mice (P = 0.74 and P = 0.50; n = 21 & 24 terminals, respectively), while the last two parameters were significantly reduced in mutants at P14 (Fig. 8C & D), (P = 0.0003 and P = 0.006, respectively). In the caudal part, however, vesicle parameters were already decreased at P7 and did not increase with age (Fig. 8F & G). Interestingly, evoked neurotransmitter release (quantal content) was not statistically different in control and mutants (WT: 10.21±0.74 and mutants: 7.48±1.22; P = 0.07; Fig. 8H) in the rostral part of the muscle at P14. These findings also show that the defect in presynaptic differentiation is not secondary to a blockade of postsynaptic differentiation.

## Discussion

The mechanisms underlying the postnatal maturation of pre- and postsynaptic specializations are finely controlled to assure reliable spatial and temporal transmission of information in and from the nervous system. At the end of the synaptic maturation process optimal juxtaposition of postsynaptic receptors and presynaptic neurotransmitter release sites is achieved. From the presynaptic side, the key elements to regulate are the position and density of proteins and organelles within the terminal. How this process is controlled, however, remains obscure. Here, we report that SMN-deficient mice present presynaptic defects in SVs, mitochondria, AZs, NFs and microtubules, as well as in neurotransmission, with a pattern compatible with a deficiency in presynaptic differentiation.

In the TVA muscle of SMN-deficient mice SVs remained clustered at P7, contrary to what happened in control mice where SVs dispersed and occupied larger areas of the terminal. In addition, SMN-deficient motor nerve terminals stopped accumulating SVs at P7, resulting in a 50% decrease in vesicles in mutants compared to controls at two week of age (Fig. 1F). This last finding was in agreement with the decrease in size of the synaptic vesicles pools found electrophysiologically at this age (Fig. 2). Strikingly, in the rostral band of the LAL these alterations appeared about a week later (Fig. 8). In the *Tibialis anterior* (TA) and in the *Extensor digitorum longus* (EDL) muscles two different ultrastructural studies, in the same SMA mouse model, also found a decrease in SVs density [35], [56]. Conversely, in the diaphragm no significant difference in vesicle number has been found [57]. Moreover, the size of the pool of SVs ready to be released (RRP) has been reported to be comparable in SMNΔ7 and littermate controls in the EDL (Ling et al., 2010), a mildly SMA affected distal muscle. However, here we found that the RRP size was decreased in the TVA in mutant mice (Fig. 2), in agreement with the ultrastructural observation of a similar decrease in docked vesicles in nerve terminals from TA muscle [35]. Therefore, there seems to exist a correlation between the degree of muscle pathology and the variability in SV content.

In the present experiments we also found a ∼50% reduction of total active mitochondrial surface in mutant presynaptic terminals (Fig. 3), with no apparent alteration in their spatial organization close to SV clusters. Previous evidence also suggests that mitochondrial defects are present in SMA nerve terminals. For example, in the TA muscle mitochondrial density is reduced by half in mutants while their morphology is normal [35], [20]. In the diaphragm, however, presynaptic mitochondria are smaller in mutants than in wild-type littermates, while no differences are found at the postsynaptic site [32]. In the TVA, the amount of Ca^2+^-dependent asynchronous neurotransmitter release during prolonged stimulation is increased, which might suggest an altered regulation of bulk [Ca^2+^] by the mitochondria [36]. Mitochondrial dysfunction has also been reported when Smn is knocked down in cultured neuronal cells, a cell model of SMA [58]. Mitochondrial defects have been demonstrated in other motor neuron diseases, including ALS [59], [60] and Spinal and Bulbar Muscular Atrophy [61].

Beside the reduction and clustering of SVs and mitochondria in SMA mutant terminals, we also found a reduction in the density of AZs (visualized with anti-bassoon antibodies), together with an alteration in their distribution (Fig. 4). This, together with the decrease in the RRP size (Fig. 2), may explain the drop in neurotransmitter release as evidenced by estimation of the quantal content [35], [36]. A smaller number of AZs might be caused by a deficiency of presynaptic P/Q- and N-type voltage-dependent calcium channels in the nerve terminal [62], [63]. In motoneurons in culture from SMN deficient embryos, N-type calcium channels have been described to be diminished at growth cones [64]. It could be of interest, therefore, to explore this possibility at motor terminals of postnatal mutant mice in the future.

It is interesting to note the parallels between the decrease in SVs, mitochondria and AZs in SMN deficient nerve terminals (Figs. 1, 3 & 4). The association between mitochondria and SVs is relevant for ATP-dependent functions such as refilling of SVs with neurotransmitter. On the other hand, the close relationship between AZs and SVs is essential for the efficient refilling of the release sites. In addition, the proximity of mitochondria to the plasma membrane may also support SV cycling. Thus, in SMN deficient terminals, the reduction of these organelles could be partially responsible for the functional impairment of the synapse. It is difficult to discern, however, whether the decrease in mitochondrial density is a consequence of the decrease of the SV pools. Other possibilities are also feasible, for example, a malfunction of the control mechanisms that maintains an appropriate pool of mitochondria within the presynaptic terminal. The interactions of mitochondria and SVs with the cytoskeleton are crucial for localization and maintenance of these organelles at their sites of action. For example, the subcellular localization (docking process) of mitochondria is likely based on F-actin filaments [65], [66], in turn regulated through the RhoA/formins pathway [67]. Finally, a deficiency in the microtubule-based anterograde transport of mitochondria, SVs and AZ precursors, is also plausible. A fault in the microtubule network itself, or in the motor proteins responsible for those cargos, may cause this phenotype. In support of this possibility, it has recently been reported that there is a decrease in polymerized tubulin at distal axons of SMN deficient terminals and less amount of mitochondria in the motor neurons [39].

For all these reasons we also studied the cytoskeleton (NFs, actin filaments, and microtubules) in SMNΔ7 motor terminals. Previously, it has been reported that NFs accumulated in motor axons and in the terminals of SMA mouse models [32], [33], [34], [35], [36]. The importance of NF accumulation in the SMA pathogenesis is difficult to determine. NF overpacking may impair axonal transport of vesicles to terminals. However, we found that SV content in nerve terminals of the TVA muscle was already reduced at one-week of age (Fig. 1F), a time at which abnormal NF accumulation was not apparent (Fig. 5E), suggesting that NF accumulation is a late event in the disease progression. In addition, we also found a marked tendency of intraterminal NFs to end in balls in SMA mutants at P14 (Fig. 5D), a sign of immaturity that suggests impairment in NF assembly and turnover.

A defect in axonal transport in SMN-deficient animal models cannot, nevertheless, be discarded. SMN has been implicated in the axonal transport of mRNA of beta-actin and other cargoes necessary for motor neuron integrity and function [19], [26]. In nerve terminals, F-actin is known to play important roles in SV recycling [55], [68] and, probably, in the tethering of SVs at AZs [69]. Therefore, even a small decrease in actin content in SMA motor terminals (Fig. 6), may affect one or more functions related to the transport or the stability of these organelles. Although this hypothesis is attractive, however, a newly generated motor neuron specific beta-actin conditional knock-out mouse does not present an altered phenotype at the NMJ [70].

Recently, it has been reported that microtubule polymerization is disrupted in Smn-deficient NSC34 cells in culture, that the amount of acetylated tubulin is about one third of controls in sciatic nerves of SMA mice and that the number of axonal microtubules per axon is reduced by 25% in mutant mice [39]. We here found that the presynaptic motor terminals of SMNΔ7 mice show a reduction and an abnormal distribution of microtubules (Fig. 7), compatible with the arrest of postnatal maturation at the presynaptic terminal. The scattering of microtubules in mutant terminals might, in turn, contribute to the delayed organization of the synaptic organelles.

In summary, our data show that SMN is essential for postnatal maturation of SVs, AZs, mitochondria and the cytoskeleton at the motor nerve terminal. We also suggest that this disruption in the presynaptic architecture might limits synaptic transmission in most affected muscles. These results, together with data from others showing delay in the maturation of the postsynaptic terminal, failure of muscle fiber growth, and a decrease in synaptic inputs to spinal motor neurons [37], [71], [72], support a possible role of SMN in neuromuscular development. Future investigations in this direction, therefore, may help to better understand the pathophysiology of this disease.

## Materials and Methods

### Mouse model

Mouse lines were kindly provided by Dr. A. Burghes. Experimental mice were obtained by breeding pairs of SMA carrier mice (Smn+/−; SMN2+/+; SMNΔ7+/+) on a FVB/N background. Identification of wild-type (WT) and SMA mice (Smn-/-; SMN2; SMNΔ7) was done by PCR genotyping of tail DNA as previously described [40]. All experiments were performed according to the guidelines of the European Council Directive for the Care of Laboratory Animals.

### Muscle preparation

Mice were sacrificed by CO_2_. The *Levator auris longus* (LAL) and *Transversus abdominis* (TVA) muscles were dissected with their nerve branches intact and pinned to the bottom of a 2 ml chamber, over a bed of cured silicone rubber (Sylgard, Dow Corning Corp.). Preparations were continuously superfused with physiological solution (in mM): 125 NaCl, 5 KCl, 2 CaCl_2_, 1 MgCl_2_, 25 NaHCO_3_ and 15 glucose, continuously gassed with 95% O_2_ and 5% CO_2_ (pH: 7.35). Electrophysiological recordings were performed at room temperature (22–23°C).

### Intracellular recording

Standard electrophysiological techniques were used to record miniature (mEPPs) and evoked end-plate potentials (EPPs) using conventional microelectrodes (R = 10-25 MΩ fill with 3 M KCl) as described previously [36]. Recordings were done in the TVA muscle from control and SMA mice at P14-15 days of age. Nerve stimulation was carried out by a suction electrode. The stimulation consisted of square-wave pulses of 0.2-0.5 ms duration and 2–40 V amplitude, at variable frequencies (0.5–20 Hz). Muscle contractions were blocked with µ-conotoxin GIIIB (2–4 µM, Alomone), a specific blocker of skeletal muscle voltage-gated sodium channels.

Quantum contents during a train were plotted against time, and fitted to a sequential model of release [42] that assumed that all release on the first shocks came from the RRP, which is depleted along an exponential time course, and that the refilling of the RRP rose sigmoidally to a plateau level. The probability of release (Pr) was calculated by dividing the first EPP quantal content by the RRP size.

### Immunohistochemistry

Whole-mount dissected muscles were incubated for 30 min in Ringer solution saturated with 95% O_2_/5% CO_2_ before fixation in 4% paraformaldehyde. Later, muscles were bathed in 0.1M glycine in PBS for 20 min, then permeabilized with 1% (v/v) Triton X-100 in PBS for 30 min and incubated in 5% (w/v) BSA, 1% Triton X-100 in PBS for 2 h. Samples were incubated overnight at 4°C with the primary antibodies of interest (see below). The following day muscles were rinsed for 1 h in PBS containing 1% Triton X-100, incubated for 1 h both with the corresponding secondary antibodies and 10 ng/ml rhodamine-BTX or BTX-Alexa 647 (BTX-A647) (Molecular Probes) and rinsed again with PBS for 90 min. Presynaptic terminals were labeled with antibodies against the vesicular acetylcholine transporter (VAChT; 1∶500, Synaptic System), the synaptic vesicle 2 protein (SV2; 1∶500, Developmental Studies Hybridoma Bank), neurofilaments (NF; 1∶750, Millipore), acetylated tubulin (1∶1000, Sigma) and Bassoon (1∶100, Stressgen). The preparations were incubated with fluorescent secondary Alexa antibodies (1∶500, Invitrogen). Finally, muscles were mounted with slowfade medium (Sigma).

### Mitochondrial labeling

Fresh tissue was incubated with Mitotracker Orange (Invitrogen), a mitochondrion-selective stain that is concentrated by active mitochondria and retained well during cell fixation, at final concentration of 400 nM. The muscle was then washed, fixed and processed for additional labeling of other organelles.

### Actin labeling

Fixed (4% PFA) and permeabilized (1% Triton X-100) muscles were incubated with the actin dye Phalloidin-Alexa647 (Invitrogen) at a working concentration of 170 nM. Later, the preparation was washed and mounted with slowfade medium.

### Image acquisition and analysis

Muscles were imaged with an upright Olympus FV1000 confocal laser scanning microscope, equipped with three excitation laser lines (argon-krypton laser with 488, 561 and 633 nm excitation lines). During image acquisition, an alternating sequence of laser pulses was used for activation of the different fluorescent probes. Images were taken using a 63x oil-immersion objective with a numerical aperture of 1.4. Images from wild-type and mutant littermate preparations were taken with similar conditions (laser intensities and photomultiplier voltages) and, usually, during the same day. For analysis, only fully occupied terminals were considered [33].

Morphometric analysis of the fluorescently labeled structures was performed offline with ImageJ (W. Rasband, National Institutes of Health, Bethesda, MD; http://rsb.info.nih.gov/ij/). Postsynaptic terminal, SVs and NFs areas were determined automatically, by finding outline masks based on brightness thresholding, from maximal projected confocal images.

All statistics given as mean ± standard error of the mean, unless stated otherwise. Differences between groups were tested using the *t*-test (2 tails).

## Supporting Information

Figure S1 **VAChT and SV2 colocalize in SMA synaptic vesicles. A & B.** Examples of WT and SMNΔ7 terminals at P14 from TVA muscles. Images are Z-stack projections. Scale bar: 5 µm. **C.** Quantification of colocalization by Pearson coefficient showed no significant difference between SMNΔ7 (*n* = 9 terminals) and WT (*n* = 4 terminals) mice (P = 0.78).
(TIF)
