# Supplementary figures and images for: SMN Requirement for Synaptic Vesicle, Active Zone and Microtubule Postnatal Organization in Motor Nerve Terminals
Source: PLoS One. 2011 Oct 12;6(10):e26164. doi: 10.1371/journal.pone.0026164 (PMC3192162; doi:10.1371/journal.pone.0026164)

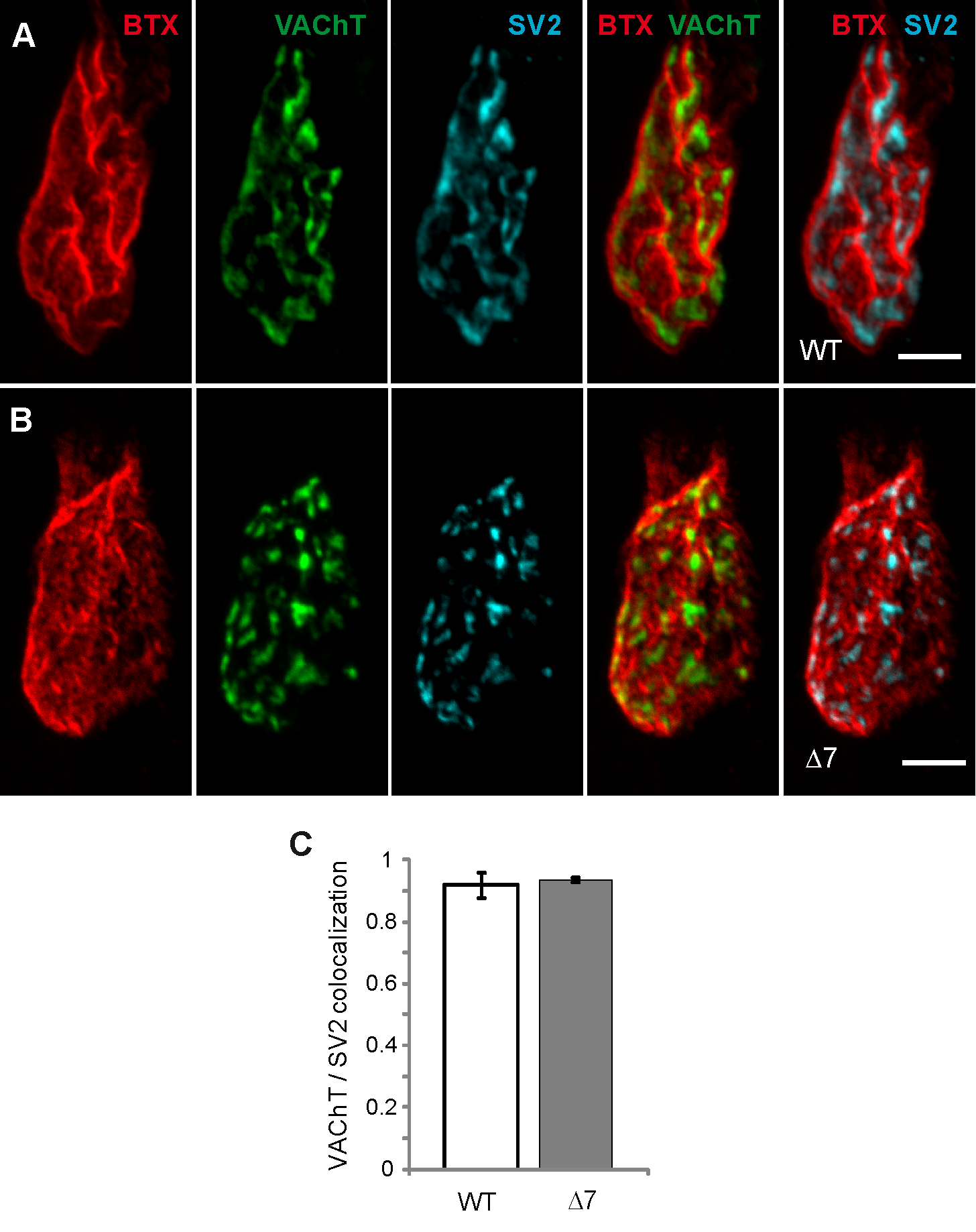

Supplement: Figure S1 — VAChT and SV2 colocalize in SMA synaptic vesicles. A & B. Examples of WT and SMNΔ7 terminals at P14 from TVA muscles. Images are Z-stack projections. Scale bar: 5 µm. C. Quantification of colocalization by Pearson coefficient showed no significant difference between SMNΔ7 (n = 9 terminals) and WT (n = 4 terminals) mice (P = 0.78). (TIF) [file pone.0026164.s001.tif]
